# The Effect of Aluminum Exposure on Reproductive Ability in the Bank Vole (*Myodes glareolus*)

**DOI:** 10.1007/s12011-016-0848-3

**Published:** 2016-09-29

**Authors:** Agata Miska-Schramm, Joanna Kapusta, Małgorzata Kruczek

**Affiliations:** Institute of Environmental Sciences, Jagiellonian University, Gronostajowa 7, 30-387 Kraków, Poland

**Keywords:** Aluminum, Bank vole, Ovarian follicles, Spermatozoa, Sperm cells

## Abstract

Human impact on the environment is steadily increasing the amounts of aluminum in the ecosystems. This element accumulates in plants and water, potentially exposing herbivores to its harmful effect. In heavily polluted sites, a decrease in the density of small rodent populations has been observed. This decline may be caused by many factors, including decreased fertility. The aim of the presented research was to determine how aluminum, administered at concentrations similar to those recorded in industrial districts (Al I = 3 mg/l, Al II = 200 mg/l), affects the reproductive abilities of small rodents. As the indicators of reproductive abilities, body weight, weight of the testes and accessory sex glands of males, and uterus weight of females were estimated. In females, the number of matured follicles (types 6, 7, and 8) was analyzed, while in males, the quantity and quality (matured, viable, swollen, motile, head abnormalities) of epididymal sperm cells were assessed. Moreover, the development of testes, measured by spermatogenic index, was determined. The model species was the bank vole. Our results have proven that aluminum impairs adult individuals’ reproductive abilities by decreasing the quality and quantity of sperm cells and by causing morphologically abnormal development of the gonads. However, no difference in male organometric parameters was found, and only in females treated with 3 mg/l Al, the uterus weight was higher than control. No differences were found in the total number of matured follicles. These results suggest that the decline in rodent numbers in industrial districts is due, at least in part, to poorer males’ reproductive abilities, resulting from exposure to aluminum contamination.

## Introduction

Soil contamination resulting in substantial concentrations of different pollutants, including metals, has been observed in plants [1], which then may be ingested by herbivores. In polluted sites, a decline in the density of rodent populations has been widely observed [2–4]. To date, there is no data published clarifying whether the decrease is due to increased mortality; other ecological processes, such as migration; or the altered reproductive abilities. The presented research addresses this question by testing the effects of aluminum on reproductive abilities of small rodents.

Aluminum has no known biological role in living organisms and may be classified as a toxic metal [5]. In vertebrates, this element may be deposited in different tissues, including the central nervous system, becoming a neurotoxin [6, 7]. This element has long been implicated in the pathogenesis of Alzheimer’s disease, but the precise mechanism of aluminum toxicity in this disease remains unknown [8]. Deposition can occur throughout the brain, as Al can cross the blood-brain barrier [9–11]. Disorders of steroidogenesis may arise through the deposition of Al in the hypothalamus and pituitary gland. However, the mechanisms of aluminum toxicity are not fully understood [12, 13].

Intraperitoneal administration of aluminum is known to decrease testosterone levels in the testes and plasma of mice, depending on the dose and duration of exposure: the reduction was much greater under treatment with a dose of 175 mg AlCl_3_/kg/day and then at 66 mg AlCl_3_/kg/day [14]. Aluminum also decreased mice serum testosterone levels and testicular and epididymal weight and significantly reduced testicular, spermatid, and epididymal sperm counts [7, 14, 15]. Aluminum accumulation in the testes has been correlated with necrosis of mice spermatids and spermatocytes, as well as reduced fertility [15]. A negative impact of aluminum on rabbit sperm cell motility and viability has been shown in vitro [16].

Knowledge of the effects of aluminum on the female reproductive system is limited. In female mice, Mohammed and collaborators [17] showed histopathological changes in the ovaries and decreased fertility, as measured by the number of pregnant females and the number of absorbed fetuses, after 12 weeks of aluminum chloride administration (dose range 1000–1400 mg/kg). Fu and collaborators [18] noted a disruption of the rat ovary structure after 64, 128, and 256 mg/kg aluminum intake, while Trif and collaborators [19] reported significant lengthening of the sexual cycle in female rats after 0.2, 0.4, and 1 mg/kg aluminum sulfate administration in the uterus.

There are no data on the impact of aluminum doses, equivalent to the environmental levels of the metal, on the reproductive system.

Plants growing on contaminated soil may accumulate substantial concentrations of different metals which then often become ingested by herbivores [20]. For animals living in polluted areas, often the only sources of water are contaminated [21]. Significant concentrations of different metals have been found in the tissues of such animals [20, 22–28]. According to Zafar and collaborators’ [29] research on laboratory rats, the first target of aluminum accumulation is bones and then the spleen, kidney, and liver. Aluminum is a chemical element abundant in the biosphere and widespread in the air (ca. 150 mg/m^3^), water (ca. 0.8 mg/l), and plants (up to 200 mg/kg) [30]. Kabata-Pendias [30] reported that exposure to 3 mg/l of aluminum did not disrupt the proper functioning of invertebrates’ internal organs. On heavily polluted sites, plants, which are the rodent’s food base, may accumulate up to 200 mg/kg aluminum from the atmosphere and water [1]; therefore, those two doses were chosen to be tested in our research. However, some species, adapted to acid soils, accumulate more than 10,000 mg/kg Al [31, 32]. The more acidic the environment, the more Al is accumulated in the plant tissues [33].

The model species chosen for these experiments is the bank vole (*Myodes glareolus*, Schreber 1780). It is the most common rodent species in Europe and Asia. Bank vole is an animal living mostly in mixed forests with rich undergrowth, thickets, meadows, and forest gaps [34–36] and foraging often in fields [37]. For many years, the bank vole was considered a polygynous species [38], but molecular techniques have revealed that females commonly mate with multiple males [39, 40] and, therefore, should be considered promiscuous. In the wild, bank vole reproductive season starts in April and lasts through late September [41]. In standard laboratory conditions, it reproduces all year long. Females give birth to one to eight pups, pregnancy lasts 18–19 days, and lactation occurs from 18 days up to 3 weeks [41]. Moreover, in the wild, it may be found in many of the contaminated areas [42]. As a small rodent, it is particularly useful as a bioindicator, which has been proven in a number of studies [24, 25, 43, 44]. Moreover, our own breeding colony originated from the wild and its reproductive biology has been very well described (for example: [45–48]). Those attributes make it a perfect model species for the presented research.

To determine the impact of aluminum on the reproductive abilities of sexually mature bank vole males, body weight and the weight of the testes and accessory sex glands were compared between animals treated with aluminum solutions and those provided with water. The quantity and quality of epididymal sperm and spermatogenic activity were also assessed. Sexually mature females’ reproductive abilities were assessed based on their body weight and uterus weight. Also, the number and type of mature follicles in females from the experimental groups were analyzed from histological slides of ovaries.

## Material and Methods

### Animals and Housing Conditions

The bank voles (*M. glareolus*, Schreber 1780) came from the laboratory colony of the Institute of Environmental Sciences, Jagiellonian University, Krakow. The original stock was obtained in 1976 from the Mammalian Research Institute of the Polish Academy of Sciences (Białowieża) and is maintained as an outbred stock colony according to the system described by Green [49]. Briefly, each generation consists of at least 22 breeding pairs; the male and female of each mating pair do not share parents or grandparents. This breeding system ensures the heterogeneity of the colony [49]. The animals were housed in polyethylene cages (40 cm × 25 cm × 15 cm) under a 14-h photoperiod (7 am–9 pm light, 9 pm–7 am dark) at 21 ± 1 °C and 60 % humidity. Wood shavings were provided as a bedding material and changed once a week. Standard pelleted chow for laboratory rodents (Labofeed H, Kcynia) and liquid in the form of deionized water or solutions of aluminum were available ad libitum.

For the study, at 19–20 days of age, the weanlings were separated from their parents and placed in clean cages. At 4 weeks of age, three to five individuals were placed in the same-sex cages. Then, both females and males were randomly divided into three experimental groups. Starting from 4 weeks of age, for 12 weeks, the animals were treated with different metal solutions or given deionized water.

### Experimental Groups

The control (C) group was provided with deionized water, Al I (3 mg/l dose)–aluminum chloride(VI) hexahydrate (AlCl_3_·6H_2_O) (AR purity grade, Avantor, Poland) at a concentration of 3 mg Al^3+^/l (1.5 mg Al^3+^/kg body mass/day), and Al II (200 mg/l dose)–aluminum chloride(VI) hexahydrate (AlCl_3_·6H_2_O) (AR purity grade, Avantor, Poland) at a concentration of 200 mg Al^3+^/l (100 mg Al^3+^/kg body mass/day).

## Reproductive Activity of Adults

### Males

#### Organometric Parameters

After cervical dislocation, 12 males from each experimental group, at 16 weeks of age, were weighed, and the paired testes, seminal vesicles, and coagulation glands were dissected out and weighed (the latter two together). Testes were placed in a fixative. Semen was collected for further analysis as described below.

#### Epididymal Sperm Evaluation

##### Preparation of Epididymal Sperm Suspension

After applying gentle pressure to each cauda epididymis with forceps, allowing epididymal sperm to pass to the vasa deferentia, the content of the latter was suspended in a 100 μl M2 medium (Sigma-Aldrich, Germany) and allowed to disperse for 2 min.

##### Epididymal Sperm Suspensions and Concentration

A 1:20 dilution of epididymal sperm suspension with the M2 medium was prepared, and the number of live sperm cells in 100 squares of a hemocytometer (Bürker chamber) was counted under a light microscope at ×400. A cover slip was placed on the sample to restrict sperm cell movement.

##### Epididymal Sperm Motility

Sperm cell motility was assessed in a hemocytometer. The proportion of motile sperm—sperm showing a progressive movement among 100 counted sperm cells—was recorded.

##### Epididymal Sperm Tail Membrane Integrity—Water Test

The integrity of the epididymal sperm tail membrane was determined in hypoosmotic swelling tests; 20 μl of epididymal sperm suspension was mixed with 120 μl distilled water on a clean glass slide. Then, the mixture was gently covered with a cover slip and incubated for 5 min at 37 °C before being examined [32]. The proportion of sperm cells showing swelling, among 100 counted sperm cells from each male, was recorded and classified as follows: swollen (sperm cells with loop on tail) and not swollen (sperm cells with straight or gently bent tail).

##### Epididymal Sperm Viability—Eosin-Y Test

The test reflects the structural and morphological integrity of the sperm membrane [50]. To assess sperm viability, 20 μl of epididymal sperm suspension was mixed with 20 μl of 0.2 % eosin-Y, incubated for 10 min at 37 °C, and smeared on a slide. The proportion of cells, with unstained heads (viable sperm cells) among 100 counted cells, was recorded.

##### Epididymal Sperm Cells Without a Cytoplasmic Droplet

In this procedure, 20 μl of epididymal sperm suspension was transferred to a slide and gently covered with a cover slip. The percentage of spermatozoa with a cytoplasmic droplet among 100 counted spermatozoa showing a progressive movement was recorded [50].

##### Epididymal Sperm Morphology

For morphological examination, a small drop of epididymal sperm suspension was smeared on a slide, air-dried, fixed in acetic alcohol (absolute alcohol/glacial acetic acid, 3:1), dehydrated in ethanol series, and stained with Papanicolaou to determine the proportions of different sperm head anomalies.

Head anomalies were classified as follows: normal (sperm with proper head morphology), class 1 (lack of the top part of the hook and anomalies in the base of head), and class 2 (lack of the hook as well as serious anomalies in the proximal part of the sperm head, with possible changes in base the of head) [32].

All above procedures were performed twice, and the mean was calculated for each individual [50].

#### Spermatogenic Index

Isolated testes were fixed in formalin, dehydrated in an ethanol series, infiltrated and embedded in paraffin, cut into 7-μm-thick cross sections, and then stained with hematoxylin and eosin and classified with light microscopy as to functional state according to the spermatogenic index [51, 52]. The spermatogenic index (SI), with values from 5 to 0, gives a measure of the seminiferous epithelium activity, with 5 representing complete spermatogenesis with abundant sperm production and 0 representing the presence of only Sertoli cells and spermatogonia; values from 1 to 4 represent incremental changes of spermatogenesis:

- IS = 0—The tubules are very small and contain only Sertoli cells and spermatogonia. A few spermatocytes are visible.
- IS = 1—There are small tubules containing only Sertoli cells, spermatogonia, and primary spermatocytes. The interstitial cell patches are very small, and most of the cell nuclei are no longer round.
- IS = 2—No elongated spermatids are present, but round spermatids still occur. Some interstitial nuclei are no longer round.
- IS = 3—There is a further reduction in the number of sperm cells and spermatids. The interstitial cell patches are much smaller, but the nuclei are still round.
- IS = 4—Spermatogenesis is complete, but elongated spermatids and sperm cells are less abundant. The interstitial cell patches are slightly smaller.
- IS = 5—The seminiferous tubules are large, and spermatogenesis is complete. The interstitial cell patches are very large, and the cell nuclei are round.

The average of two groups of ten seminiferous tubules situated in the center of the testicular cross section was taken as the spermatogenic index estimate.

### Females

#### Organometric Parameters

After cervical dislocation, 12 females from each experimental group, at 16 weeks of age, were weighed, after which the uterus was removed and weighed. Ovaries were also dissected and placed in a fixative for further analysis.

#### Ovarian Follicle Assessment

Isolated ovaries were fixed in Boinea’s solution, dehydrated in an ethanol series, infiltrated and embedded in paraffin, cut into 6-μm-thick cross sections, and then stained with hematoxylin and eosin and analyzed under a light microscope to classify the different stages. The follicles in ovaries from the females in all experimental groups were classified as type 6 (diameter 355.06–417.99 μm), type 7 (diameter 526.58–594.67 μm), or type 8 (diameter 715.78–867.39 μm) according to Pedersen and Peters [53]. Follicle diameter was determined using ImageJ 1.48k (National Institutes of Health, USA) software. The sum of each type of follicle, in an individual, was recorded.

### Statistical Analysis

The following statistical tests were used to analyze the data: one-way ANOVA for morphological parameters, sperm cell parameters, spermatogenic index, and number and type of ovarian follicles, and post hoc Tukey’s test following one-way ANOVA to test the significance of differences between means.

All procedures employed STATISTICA v. 10. All data are presented as means ± SE. The level of statistical significance was deemed to be *p* < 0.05.

## Results

### Male Organometric Parameters and Sperm Evaluation

#### Organometric Parameters

The results summarized in Table 1 show that aluminum did not affect the morphological parameters of adult males. There were no significant differences in body weight (g) between control and Al I males, between control and Al II males, or between Al I and Al II males (*p* = 0.38, NS) (NS = not significant). Similarly, weight of the testes (*p* = 0.7, NS) and accessory sex gland (mg; *p* = 0.28, NS) did not significantly differ between control and Al I males, between control and Al II males, or between Al I and Al II males.

**Table 1:** Organometric parameters of bank vole males treated with two aluminum solutions (Al I = 3 mg/l and Al II = 200 mg/l) or deionized water (C = 0 mg/l)

	Experimental group				
	C	Al I	Al II	*F*_(2,33)_	*p*
Body wt (g)	27.5 ± 1.1	28.3 ± 1.2	29.7 ± 1.1	1.01	NS
Testes wt (mg)	763.2 ± 33.4	780.4 ± 18.7	795.6 ± 27.2	0.36	NS
Accessory sex gland wt (mg)	351.9 ± 25.1	362.8 ± 25.2	412.8 ± 33.1	1.34	NS

Means ± SE

#### Epididymal Sperm Evaluation

**Fig. 1:**
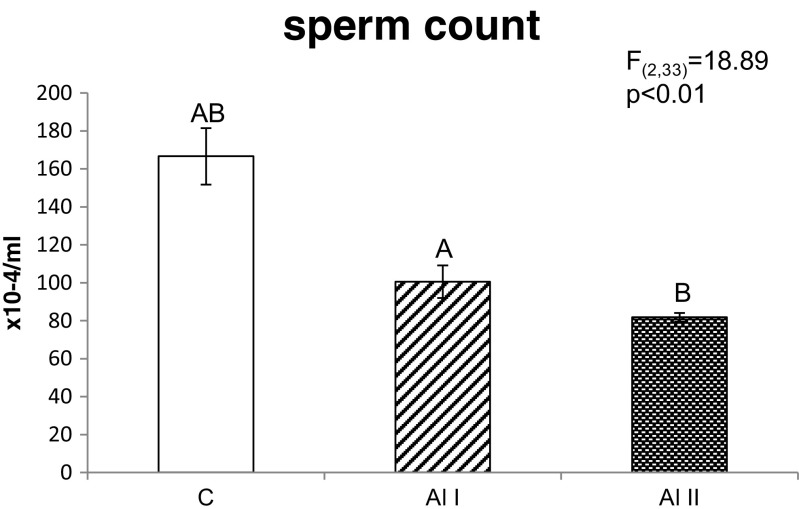
Sperm counts of adult bank vole males treated with two aluminum solutions (*Al I* = 3 mg/l and *Al II* = 200 mg/l) or deionized water (*C* = 0 mg/l). Means bearing *the same letter* differ significantly; *A*, *B p* < 0.01. Means ± SE

**Fig. 2:**
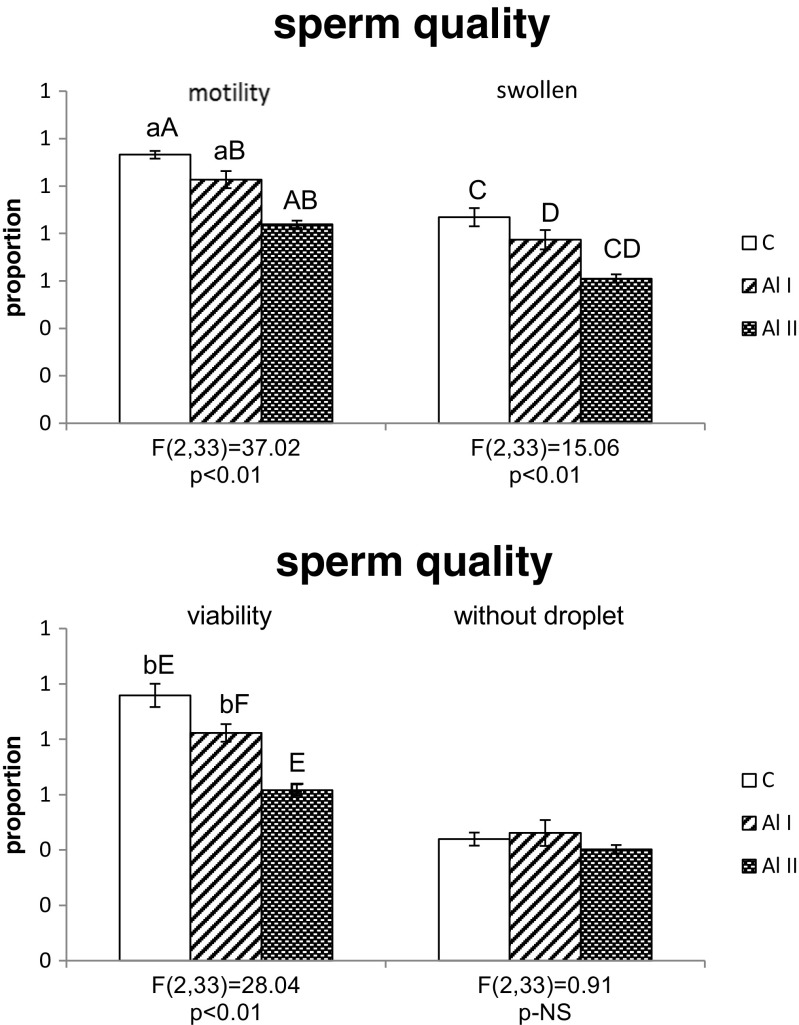
Proportion of swollen, viable, motile, and dropletless sperm cells from bank vole males treated with two aluminum solutions (*Al I* = 3 mg/l and *Al II* = 200 mg/l) or deionized water (*C* = 0 mg/l). Means bearing *the same letter* differ significantly; *A*–*F p* < 0.01; *a*, *b p* < 0.05. Means ± SE

As shown in Figs. 1 and 2, exposure to aluminum lowered sperm quantity and quality. Sperm counts were significantly lower in males treated with 200 and 3 mg/l Al than in control males. There were no significant differences in sperm count between Al I and Al II males (*p* = 0.39, NS). The proportion of motile sperm cells was lower in Al II males than in both the control and Al I animals. Al I males also had a lower proportion of motile sperm cells than control males (*p* < 0.01). The proportion of swollen sperm cells was lower in Al II males than in both the control and Al I males, but there were no differences in the proportion of swollen sperm cells between control and Al I males (*p* = 0.14, NS). As shown in Fig. 2, the proportion of viable sperm cells was lower in Al II males than in control and Al I males. Males from the Al I group had also a lower proportion of viable sperm cells than control individuals (*p* < 0.01). There were no differences in the proportion of mature sperm cells without a droplet between males from the control, Al I, and Al II groups (*p* = 0.42, NS; Fig. 2). As presented in Table 2, Al II males had a significantly higher proportion of abnormal sperm heads than both the control and Al I males (*p* < 0.01). There were no differences in the total proportion of abnormal sperm heads between males given deionized water and those receiving 3 mg/l aluminum (*p* = 0.11, NS; Table 2). There were significant differences in the proportions of both classes of abnormal sperm heads between males from all experimental groups (Table 2). The proportion of class 1 abnormal sperm heads was the highest in Al II males and higher in control and Al I animals, and the same relations were found for the proportion of class 2 abnormal sperm heads (*p* < 0.01). There were no significant differences in the proportion of class 1 (*p* = 0.13, NS) as well as class 2 (*p* = 0.1, NS) abnormal sperm heads between males from the control and Al 1 groups.

**Table 2:** Proportion of sperm cell head abnormalities and spermatogenic index in bank vole males treated with two aluminum solutions (Al I = 3 mg/l and Al II = 200 mg/l) or deionized water (C = 0 mg/l)

	Experimental group				
	C	Al I	Al II	*F*_(2,33)_	*p*
Total abnormalities	0.22^A^ ± 0.01	0.29^B^ ± 0.03	0.43^AB^ ± 0.03	17.77	<0.01
Class 1	0.13^A^ ± 0.02	0.19^a^ ± 0.02	0.28^Aa^ ± 0.02	12.82	<0.01
Class 2	0.15^A^ ± 0.02	0.21^B^ ± 0.03	0.32^AB^ ± 0.02	15.23	<0.01
Spermatogenic index	4.8^A^ ± 0.1	4.6^B^ ± 0.0	3.7^AB^ ± 0.0	216.54	<0.01

Means bearing the same letter differ significantly; means ± SE

^A,B^ *p* < 0.01; ^a^ *p* < 0.05

#### Spermatogenic Index

The highest dose of aluminum (200 mg/l, Al II) had a negative effect on spermatogenesis stage as measured by the spermatogenic index (Table 2). Al II animals had a lower spermatogenic index than both control and Al I males (*p* < 0.01). The spermatogenic index of control and Al I males did not differ significantly (*p* = 0.07, NS).

### Female Organometric Parameters and Ovarian Follicle Evaluation

#### Organometric Parameters

As shown in Table 3, there were no differences in body weight (g) between control and Al I females, between control and Al II females, or between Al I and Al II females (*p* = 0.89, NS). Al I females had a higher uterus weight (mg) than control individuals (*p* < 0.05). There were no differences in uterus weight between control and Al II females (*p* = 0.17, NS) or between Al I and Al II females (*p* = 0.38, NS).

**Table 3:** Organometric parameters of bank vole females treated with two aluminum solutions (Al I = 3 mg/l and Al II = 200 mg/l) or deionized water (C = 0 mg/l)

	Experimental group				
	=	Al I	Al II	*F*_(2,33)_	*p*
Body wt (g)	23.5 ± 1.1	21.9 ± 1.0	21.9 ± 0.6	0.36	NS
Uterus wt (mg)	63.8^a^ ± 10.2	99^a^ ± 6.3	79.4 ± 8.0	0.02	<0.05

Means bearing the same letter differ significantly; means ± SE

^a^ *p* < 0.05

#### Ovarian Follicle Evaluation

As seen in Table 4, there were no differences in the total number of ovarian follicles (sum of types 6, 7, and 8) between the females from all experimental groups (*p* = 0.48, NS). The same was true for follicle types 6 (*p* = 0.44, NS) and 8 (*p* = 0.45, NS) which were analyzed separately. The number of type 7 follicles was higher only in Al I than in Al II females (*p* < 0.01); there were no significant differences in the proportion of type 7 follicles between control and Al I females (*p* = 0.07, NS) or between control and Al II females (*p* = 0.52, NS).

**Table 4:** Number of ovarian follicles in bank vole females treated with two aluminum solutions (Al I = 3 mg/l and Al II = 200 mg/l) or deionized water (C = 0 mg/l)

	Experimental group				
	C	Al I	Al II	*F*_(2,33)_	*p*
Total	12.8 ± 1.6	14.3 ± 1.5	11.9 ± 0.9	0.76	NS
Type 6	5.0 ± 0.9	5.0 ± 0.9	5.1 ± 0.9	0.83	NS
Type 7	3.6 ± 0.6	5.5^A^ ± 0.7	2.7^A^ ± 0.5	6.06	<0.01
Type 8	4.2 ± 0.8	3.8 ± 0.7	2.9 ± 0.7	0.81	NS

Means bearing the same letter differ significantly; means ± SE

^A^ *p* < 0.01

## Discussion

In male bank vole, aluminum ingestion did not affect body weight or any other tested morphological parameter. The literature reports various findings on the effects of aluminum on the reproductive parameters of males of different species. Exposure to aluminum within a concentration range of 3–200 mg/l seems to have no effect on the body, testes, and epididymal weights of other adult rodents [54]. Only much higher aluminum concentrations significantly reduced the weight of those organs in mice [55] and rats [56]. These can be explained by the finding that aluminum concentrations higher than 200 mg/l are correlated with lower levels of testosterone [57, 58], the main androgen controlling reproductive tissue development in males.

We did not find any effect of aluminum on male reproductive organ weights but did show a decline of spermatogenic activity (SI) inversely proportional to the aluminum concentration. Other researchers have found similar effects of aluminum in mice [59] and rats [60, 61]. Seminiferous epithelium activity is a key factor in spermatogenesis [62] and, along with sperm count and sperm quality, is crucial to the successful fertilization. Sperm quality and quantity, as assessed by its motility, sperm tail morphology, viability, and head abnormalities, were curtailed by aluminum in our experiment. Only sperm maturation, measured as the proportion of sperm cells without a cytoplasmic droplet, was not affected by the aluminum ingestion, probably because the aluminum does not affect the morphology of the epididymis, where final sperm maturation takes place [62, 63]. However, under Al exposure, sperm cells with abnormal head morphology were produced, and this process also takes place and is regulated in the epididymis [64]. Lower sperm counts and altered sperm parameters under aluminum exposure have also been found in humans [65, 66], rats [54], and rabbits [16, 67]. Sun and collaborators [58] found lower testosterone levels in male rats exposed to 256.72 mg/kg Al, so it is possible that testosterone disorders occurred in the 200 mg/l Al treatment. Spermatogenic activity, spermatogenesis, and spermiogenesis are mainly under the control of testosterone [68]. Their negative effect on SI, sperm quality, and quantity may be explained by the effect of 200 mg/l dose, disturbing testosterone homeostasis, but two other mechanisms may also play a role. Guo and colleagues [69]suggested that aluminum induces production of nitrogen monoxide (NO), a suppressor of circulating and testicular testosterone. Alternatively, Zhu and collaborators [70] suggested that the main reason for reduced spermatogenesis in male rats was a decline in testicular enzyme activity and an imbalance in the concentrations of other trace elements (Zn, Fe, Cu) in the testes. Further investigations should shed more light on those mechanisms.

Aluminum is a non-physiological element considered to be a potent neurotoxicant [71]. It can cross the blood-brain barrier and may be deposited in brain tissue [9–11]. It deposits in most areas of the brain (the cerebellum, ventral midbrain, cortex, hippocampus, and striatum), depending on the form of exposure: a greater and more significant increase was noted in the group of rats receiving aluminum via an intraperitoneal administration, then in rats receiving aluminum via an oral administration [9, 72, 73]. The neurodegenerative effects of the intracisternal injection of Al included the formation of intraneuronal neurofilamentous aggregates, oxidative stress, and apoptosis [74]. Verstraeten and collaborators [75] in their review about Al and molecular mechanisms of brain toxicity, indicated that Al neurotoxicity is not caused by a single alteration, but it is a result of adverse effects at multiple cellular levels. Barabasz and collaborators [76] suggested that neurotoxic effects of aluminum result in the displacement of magnesium ions in ATP, which causes changes in the functioning of all enzymes utilizing ATP as a substrate. Aluminum may also play a role on the hormonal level, by interfering with some of the neurohormonal pathways, like the serotonin system [77].

Aluminum’s influence on the brain tissue potentially may also play a role in the interruption of reproductive processes [78]. Our experiments showed almost no influence of aluminum on adult females’ reproductive abilities. Females treated with 3 mg/l Al had an increase of uterus weight as compared to control females. That increase was accompanied by an increased number of type 7 ovarian follicles in the females treated with 3 mg/l Al. Research on adult rat females employing aluminum doses three and six times smaller than our lower dose showed no effect of aluminum on body and uterus weights [79, 80]. Much higher Al concentrations (1000–1400 mg/kg) reduced female body weight, reduced absolute uterus weight, and caused histological changes in ovarian sections [17]. It may be that the concentrations we applied, which are near-normal, though at levels found in polluted districts, were too low to cause significant ovarian disorders. However, this assumption would not be coherent with overall obtained result. Both sexes were exposed to the same aluminum concentration, but in males, the damage caused by aluminum on the reproductive system is more visible than that in females. This sex-specific difference may lay in the detoxification systems.

The liver is considered to be the main detoxification target organ. It is characterized by sexually dimorphic gene expression translating into sex-specific differences in xenobiotic, for example aluminum and metabolism, with distinct responses of males and females to environmental challenges [81, 82]. In mice, examples of sex-dependent genes include the male-predominant cytochrome P450 Cyp2d9, which encodes testosterone 16-a-hydroxylase inactivating the main male sex hormone, and the female-predominant Cyp2a and Cyp2b genes involved in xenobiotic metabolism [82]. Kalthoff and collaborators [83] indicated that transcriptional regulation of human uridine diphosphate glucuronosyltransferase genes (UGT1A), important hepatogastrointestinal detoxification enzymes for xenobiotics, is also gender-specific. In response to environmental challenges, on a molecular level, males display a higher predisposition than females for liver abnormalities [84, 85]. Therefore, liver handicap in males may result in a more rapid reproductive biology decline caused by aluminum intoxication. Moreover, Gómez and collaborators [86] have proven that tissue Al retention patterns may be significantly altered and are also depending on the age at which Al exposure occurs.

A properly functioning reproductive system and well-developed reproductive organs are not the only key ingredients of reproductive success; sexual behavior is important as well. Aluminum concentrations, similar to those applied in our experiments, are correlated with changes in non-reproductive rodent behavior [87–90]; it is reasonable to suggest that aluminum may also modify sexual behavior. Indeed, Abu-Taweel and collaborators [57] found a significant decrease of social contacts and sexual behavior after aluminum application, but they used higher doses (300 and 600 mg/kg) than those in our experiment. Our pilot behavioral research did not indicate changes in rodent sexual behavior under aluminum exposure, except for fewer aggressive approaches, in a preference test by females to males treated with 200 mg/l Al than, to control males. Because the aggressiveness is considered a part of bank vole sexual behavior [91] and might be correlated with an aluminum-induced decrease of libido [16], more behavioral research in this field should be performed. Additionally, to extrapolate the obtained results into a natural environment, it would be required to run similar types of measurements on wild-caught voles from Al-polluted sites and to couple such measurements with population studies, to determine whether there are effects on reproductive success and juvenile recruitment into the population.
